# High-fat Western diet alters crystalline silica-induced airway epithelium ion transport but not airway smooth muscle reactivity

**DOI:** 10.1186/s13104-023-06672-w

**Published:** 2024-01-03

**Authors:** Janet A. Thompson, Michael L. Kashon, Walter McKinney, Jeffrey S. Fedan

**Affiliations:** 1 Health Effects Laboratory Division, National Institute for Occupational Safety and Health, Morgantown, WV 26505 USA; 2 Pathology and Physiology Research Branch, National Institute for Occupational Safety and Health, 1000 Frederick Lane, Morgantown, WV 26508 USA

**Keywords:** Western diet, Obesity, Ion transport, Airway hyperreactivity, Epithelium, Silica

## Abstract

**Objectives:**

Silicosis is an irreversible occupational lung disease resulting from crystalline silica inhalation. Previously, we discovered that Western diet (HFWD)-consumption increases susceptibility to silica-induced pulmonary inflammation and fibrosis. This study investigated the potential of HFWD to alter silica-induced effects on airway epithelial ion transport and smooth muscle reactivity.

**Methods:**

Six-week-old male F344 rats were fed a HFWD or standard rat chow (STD) and exposed to silica (Min-U-Sil 5^®^, 15 mg/m^3^, 6 h/day, 5 days/week, for 39 d) or filtered air. Experimental endpoints were measured at 0, 4, and 8 weeks post-exposure. Transepithelial potential difference (V_t_), short-circuit current (*I*_SC_) and transepithelial resistance (R_t_) were measured in tracheal segments and ion transport inhibitors [amiloride, Na^+^ channel blocker; NPPB; Clˉ channel blocker; ouabain, Na^+^, K^+^-pump blocker] identified changes in ion transport pathways. Changes in airway smooth muscle reactivity to methacholine (MCh) were investigated in the isolated perfused trachea preparation.

**Results:**

Silica reduced basal *I*_SC_ at 4 weeks and HFWD reduced the *I*_SC_ response to amiloride at 0 week compared to air control. HFWD + silica exposure induced changes in ion transport 0 and 4 weeks after treatment compared to silica or HFWD treatments alone. No effects on airway smooth muscle reactivity to MCh were observed.

## Introduction

Over 35% of the American adult population have metabolic dysfunction (MetDys) [1], yet there remains a gap in our understanding of the associated risks of MetDys and susceptibility to hazardous occupational exposures. MetDys is associated with impaired lung function [2, 3], asthma [4, 5], COPD [6, 7], and restrictive lung diseases [5]. Biomarkers of MetDys were predictors for severe lung injury in 9/11 first responders [8–11] and having ≥ 3 MetDys conditions increased the risk of developing airway hyperreactivity [12]. Previously, we found that HFWD-induced MetDys exacerbated crystalline silica-induced lung injury [13] and altered silica-induced serum inflammatory cytokines, adipokines, and arterial blood flow [14] in F344 rats. These studies established that MetDys can increase worker susceptibility to hazardous inhalation exposures.

Silicosis is an irreversible progressive lung disease caused by occupational inhalation exposure of respirable crystalline silica dust [15, 16]. Respirable-sized crystalline silica particles are highly reactive, deposit deep in the lung, exert toxicity when coming in contact with epithelial cell surfaces, and become phagocytized by alveolar macrophages (AM). Phagocytized silica particles are transported to the AM lysosome, where they interact with the lysosomal membrane, causing content leakage into the cytoplasm, activation of the NLRP3 inflammasome, and eventual cell death [17]. The release of caustic AM contents along with silica particles back into the alveolar space, to be phagocytosed by other AMs, creates a positive feedback loop that results in progressive pulmonary inflammation. Pro-inflammatory cytokines, including TNF-α, IL-1β and TGF-β, released by AMs activate alveolar fibroblasts to deposit collagen and elastin, thus contributing to the development of pulmonary fibrosis [18–21].

While pathological mechanisms of silicosis of the lung are well described, there is less understanding of the impact of silica exposure on the function of airway epithelium. Recent studies found that silica exposure induced genotoxicity of the nasal epithelium in Italian silica-exposed workers [22], and in vitro silica induces DNA damage in bronchial epithelial cells through the activation of the autotaxin-lysophosphatidic acid axis [23]. Yet another study found that mice exposed to crystalline silica particles by instillation exhibited airway epithelial cell cilia loss, rearrangement of microtubules, disruption of the axoneme, and increased MUC5B production [24]. In this study we investigate effects on airway epithelial ion transport because it is responsible for maintaining airway surface liquid (ASL) height and composition, which is critical for clearance of particles and pathogens via the mucociliary escalator. Perturbations of airway ion transport can increase mucus viscosity and impair mucociliary clearance [25, 26] and silicosis is associated with an increased risk of tuberculosis in humans [27].

Bronchial hyperresponsiveness (BHR) is not known to be associated with silicosis but is associated with obesity, hyperinsulinemia, and impaired glucose metabolism [28, 29]. BHR can also result from exposures to irritants or sensitizers and is a hallmark of occupationally-induced asthma [30]. In animal studies, two mechanisms of hyperinsulinemia-induced BHR have been identified: (1) insulin inhibition of neuronal M2 muscarinic receptors causing increased release of acetylcholine (ACh) from airway parasympathetic nerves [31, 32]; and (2) insulin stimulation of brain stem signaling pathways leading to increased airway reactivity [33]. Previously, we found that HFWD-consumption increased serum insulin at 0 and 4 weeks and HFWD + SIL increased serum insulin at  4 weeks compared to STD + AIR controls [14]. Therefore, we hypothesized that HFWD-induced hyperinsulemia, in both the air and silica exposed groups, could result in changes to airway smooth muscle reactivity.

The novel aims of this study were to determine whether HFWD consumption alters silica-induced responses in the non-ventilatory pulmonary function parameters of epithelial ion transport and airway smooth muscle reactivity. In addition, we characterize the effects of silica exposure and HFWD-consumption independently on these parameters.

## Materials and methods

### Animals and diet

**Fig. 1:**
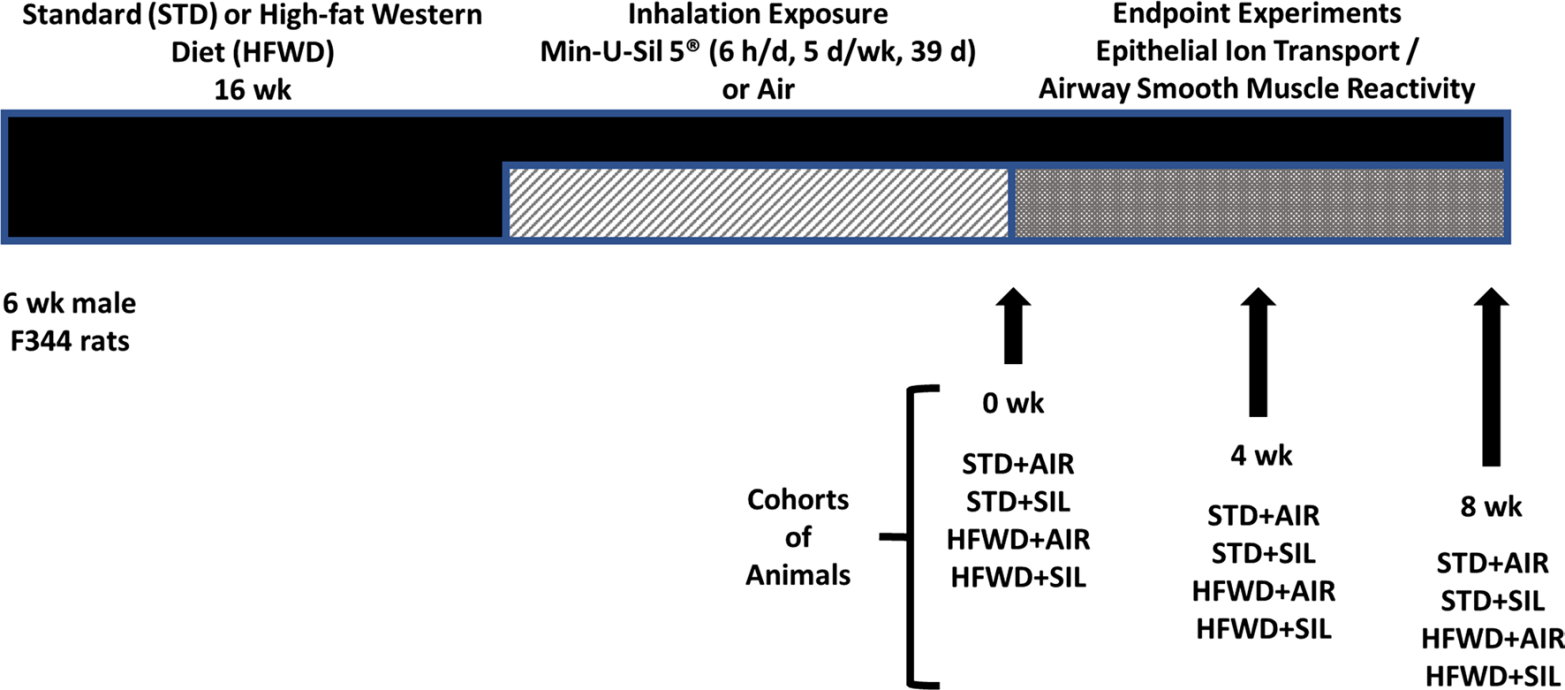
Experimental design for HFWD-induction of MetDys, silica-inhalation exposure and endpoint experiments. Schematic describes the design for single endpoint experiments using separate cohorts of animals (n = 8 for each group). Reproduced from Thompson et al. [14] with modifications

Six-week-old male Fischer (CDF) rats (F344/DuCrl) obtained from Charles River Laboratories, Inc. (Wilmington, MA), were divided into two dietary groups and fed either a commercially available “Western” diet (high-fat Western diet, HFWD; 45% fat Kcal, sucrose 22.2% by weight) or a standard rat chow (standard diet, STD; fat 6.2% by weight, sucrose-free) for the duration of the study, including silica exposure and post-exposure time points. Following 16 weeks of diet consumption, the animals were then exposed to silica (Min-U-Sil 5^®^) for 6 h/d, 5 d/week, 39 d or filtered air. The experimental design for this study is illustrated in Fig. 1, animal usage is shown in Table 1.

**Table 1:** Animal Usage

	Number of animals
Ion transport study	
STD + AIR 0 week	8
STD + SIL 0 week	8
HFWD + AIR 0 week	8
HFWD + SIL 0 week	8
STD + AIR 4 weeks	8
STD + SIL 4 weeks	8
HFWD + AIR 4 weeks	8
HFWD + SIL 4 weeks	8
STD + AIR 8 weeks	8
STD + SIL 8 weeks	8
HFWD + AIR 8 weeks	8
HFWD + SIL 8 weeks	8
Smooth muscle study	
STD + AIR 0 week	8
STD + SIL 0 week	8
HFWD + AIR 0 week	8
HFWD + SIL 0 week	8
STD + AIR 4 weeks	8
STD + SIL 4 weeks	8
HFWD + AIR 4 weeks	8
HFWD + SIL 4 weeks	8
STD + AIR 8 weeks	8
STD + SIL 8 weeks	8
HFWD + AIR 8 weeks	8
HFWD + SIL 8 weeks	8
Total animals	192

Each animal was a single unit. An n = 8 deemed sufficient to observe significant differences between groups using a power analysis conducted by a NIOSH staff statistician (MLK). There was a total of 96 animals used for each experiment and 192 for the entire study

All studies were conducted in facilities accredited by AAALAC International and were approved by the Center for Disease Control / National Institute for Occupational Safety and Health / Health Effects Laboratory Branch (CDC/NIOSH/HELD) Institutional Animal Care and Use Committee (protocol 18-011) and in compliance with the PHS Policy on Humane Care and Use of Laboratory Animals and the NIH Guide for the Care and Use of Laboratory Animals. All animals were free of viral pathogens, parasites, mycoplasm, *Heliobacter* and cilia-associated respiratory bacillus. Animals were acclimated for one week upon arrival and housed in ventilated micro-isolator units supplied with HEPA-filtered laminar flow air (Lab Products OneCage; Seaford, DE), with Teklad Sanichip and Teklad Diamond Dry cellulose bedding (or Shepherd Specialty Paper’s Alpha-Dri cellulose; Shepherd Specialty Papers; Watertown, TN). They were provided filtered tap water and autoclaved Teklad Global 18% protein rodent diet (Harlan Teklad; Madison, WI) ad libitum. Rats were housed in pairs under controlled light cycle (12 h light/12 h dark) and temperature (22–25 °C) conditions.

### Silica exposure

Crystalline silica (Min-U-Sil 5^®^; Berkeley Springs, WV; “SIL”), aerosolized using an automated exposure system [34]. Particles had median aerodynamic diameter of 1.6 µm and geometric standard deviation of 1.6, particle aerodynamic mass distribution was measured using a 10-stage impactor (MOUDI, model 110-R) in series with a nano-impactor (MOUDI, model 115). The samples were taken from inside the inhalation exposure chamber a few inches above the animal cage rack. A normal curve was fitted to the MOUDI size data for MMAD and GSD determination. Target silica concentration (15 ± 1 mg/m^3^) was monitored using a DataRAM 4 Particulate Monitor (Thermo Fisher Scientific; Waltham, MA) and controlled within the exposure chamber in real time. Daily average aerosol concentrations were also determined gravimetrically with 37 mm cassettes containing Teflon filters at the end of each exposure period to verify and calibrate the DataRAM readings. Animals were exposed using whole-body exposure chambers and control animals were exposed to filtered air and handled identically.

### Measurement of airway epithelial ion transport ex vivo

The Ussing chamber was employed to measure changes in ion transport across the tracheal epithelium. Animals were euthanized by bleeding following i.p. 300–500 mg/kg pentobarbital, tracheal tissue was removed and mounted in a Ussing chamber system (Physiologic Instruments, Inc; Reno, NV) containing modified Krebs–Henseleit solution (MKHS; 113 mM NaCl, 4.8 mM KCl, 2.5 mM CaCl_2_, 1.2 mM KH_2_PO_4_, 1.2 mM MgSO_4_, 25 mM NaHCO_3_, and 5.7 mM glucose) saturated with 95% O_2_/5% CO_2_ at 37 °C. The tissue was stabilized under open circuit conditions for measurement of transepithelial voltage (V_t_, mV) followed by application of a 0-mV voltage clamp and delivery of 1 mV pulses (5 s duration, 55 s interval). Short-circuit current (*I*_SC_) was recorded (BioPac Systems; Goleta, CA) from which transepithelial resistance (R_t_) was calculated using Ohm’s law. To identify changes in ion transport the following ion channel inhibitors were used: Na^+^ channel inhibitor amiloride (3.5 × 10^–5^ M, apical bath), Cl‾ channel inhibitor 5-nitro-2-(3-phenylpropyl-amino) benzoic acid (NPPB; 10^–4^ M, apical bath), and Na^+^,K^+^-pump inhibitor ouabain (10^–4^ M, basolateral bath). Basal values and agent-induced responses from basal value were calculated.

### Measurement of airway smooth muscle reactivity ex vivo

The isolated perfused trachea (IPT) preparation was used to measure changes in airway smooth muscle reactivity and epithelial barrier function [35, 36]. Animals were euthanized (see above) and a 25 mm segment of trachea was removed and mounted on a tracheal perfusion holder which was bathed in and perfused with MKHS, equilibrated for 1 h and washed at 15 min [35, 36]. Methacholine (MCh), a muscarinic receptor agonist, concentration–response curves were obtained by addition of stepwise increases of MCh concentrations added to the extraluminal (EL) bath, followed by a 90 min wash period at 15 min intervals, and then intraluminal (IL) additions of MCh. MCh concentration–response curves were derived from increases in inlet minus outlet pressure differences (cm H_2_0) and normalized as a percentage of the maximal contractile response. EC50 values were calculated for each individual preparation.

### Statistical analysis

Analyses were carried out using the proc mixed procedure in SAS version 9.4 or JMP version 13.2. A four-way mixed model analysis of variance (diet x treatment x time x MCh concentration) was performed with repeated measures on MCh. EC50 values were calculated for each animal and analyzed with a three-way ANOVA (diet x treatment x time). Values for EC50s were log-transformed to meet the assumptions of the analysis. Ussing chamber data were analyzed using mixed model three-way ANOVAs at each time point (diet x treatment x agent), with animal treated as a random variable to account for the multiple agents. Pairwise comparisons for all analyses were derived from the overall analysis using Fishers LSD. All differences were considered significant at P < 0.05. All values are presented as means ± SEM.

## Results

**Fig. 2:**
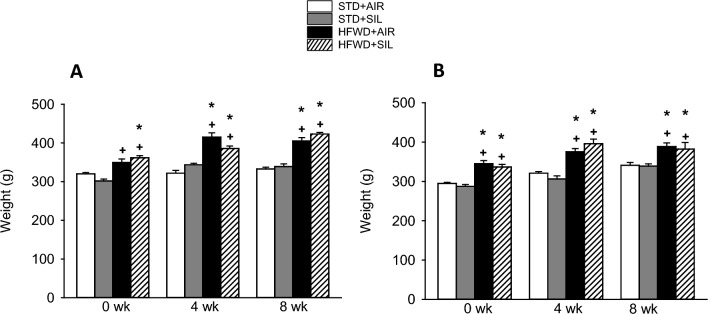
Effects silica-inhalation, HFWD-consumption, and combined exposure on body weight in animals used for **A** IPT and **B** Ussing experiments. HFWD-consumption significantly increased animal body weight at all time points compared to STD control groups regardless of silica exposure; silica-inhalation had no significant effect on body weight. *P* < 0.05. * Indicates significance compared to STD + AIR group. ^**+**^ Indicates significance compared to STD + SIL group. For IPT experiments n = 8, 8, and 5–8 at 0, 4, and 8 weeks, respectively. For Ussing experiment n = 6–8, 8, and 8 at 0, 4, and 8 weeks, respectively

Animals fed a HFWD had increased body weight compared to STD groups, but silica had no effect on body weight regardless of diet (Fig. 2). Our previous studies describe the interactions between HFWD consumption and silica exposure including HFWD exacerbation of silica-induced pulmonary inflammation and fibrosis [13], reduction of silica-induced serum cytokines, alteration of serum adipokine levels, and changes in tail arterial blood flow and pulse [14].

## Airway epithelial ion transport

**Fig. 3:**
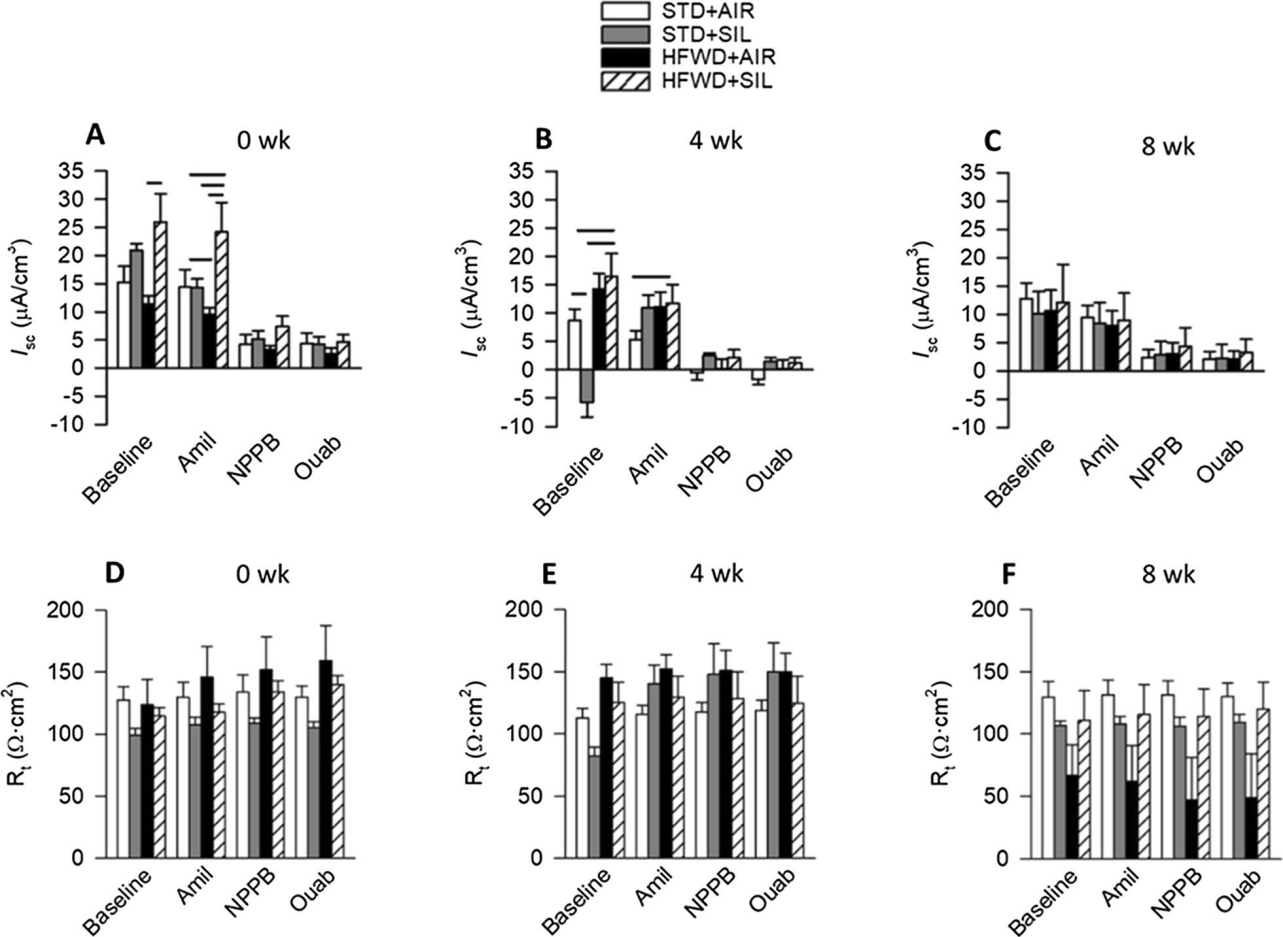
Effects of silica-inhalation, HFWD-consumption, and combined exposure on bioelectric responses to ion transport inhibitors. Shown are basal and inhibitor-induced epithelial *I*_SC_ responses at **A** 0, **B** 4, and **C** 8 weeks post-exposure. Basal and agent-induced epithelial R_t_ responses at **D** 0, **E** 4, and **F** 8 weeks. Silica reduced basal *I*_SC_ at 4 weeks (**B**). HFWD reduced *I*_SC_ responses to amiloride at 0 week (**A**). HFWD + SIL increased basal *I*_SC_ and *I*_SC_ responses to amiloride at both 0 week (**A**) and 4 weeks (**B**). Solid lines indicate significant differences between different exposure groups at a given time point. *P* < 0.05. n = 6–8, 7–8, 3–8 at 0, 4, and 8 weeks, respectively

**Fig. 4:**
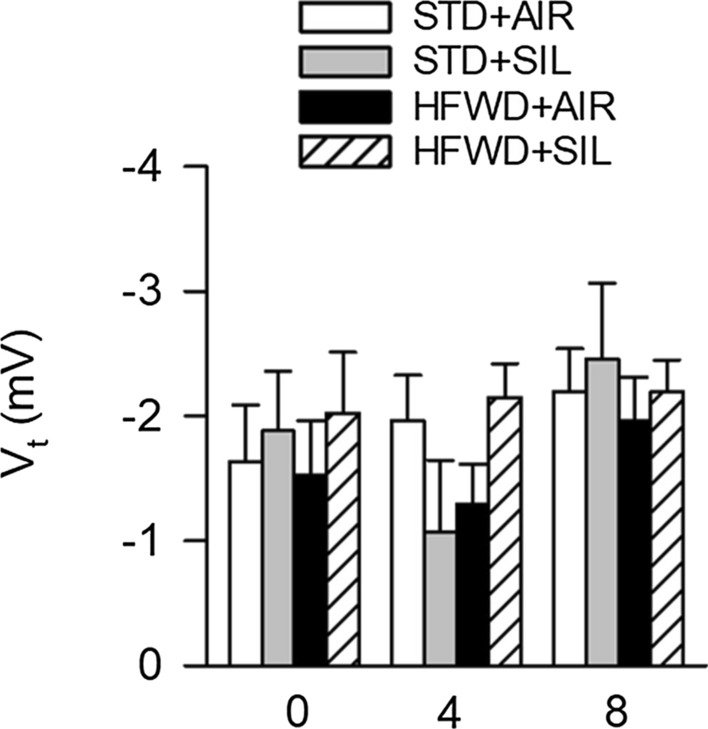
Effects of silica-inhalation, HFWD-consumption, and combined exposure on V_t_. There was no effect of silica, HFWD or combined HFWD + SIL exposure on basal V_t_. *P* < 0.05. n = 6–8, 7–8, 3–8 at 0, 4, and 8 weeks, respectively

HFWD reduced the *I*_SC_ response to amiloride at 0 week (Fig. 3A) compared to the STD + AIR, and silica reduced basal *I*_SC_ at 4 weeks (Fig. 3B) compared to the STD + AIR. HFWD + SIL increased basal *I*_SC_ compared to the HFWD + AIR and increased the *I*_SC_ response to amiloride at 0 week compared to all other groups (Fig. 3A); at 4 weeks HFWD + SIL exposure increased basal *I*_SC_ compared to the STD + AIR and STD + SIL and increased the *I*_SC_ response to amiloride compared to STD + AIR (Fig. 3B). Changes in ion transport by silica were not accompanied by or explained by changes in R_t_ (Fig. 3D, E, F), thereby ruling out altered paracellular ion transport. There were no differences in ion transport between groups at 8 weeks post-exposure (Fig. 3C). The increase in *I*_SC_ in response to amiloride following HFWD + silica exposure is indicative of increased Na^+^ ion transport across the epithelium. There was no effect of diet or silica on V_t_ (Fig. 4).

### Airway smooth muscle reactivity

**Fig. 5:**
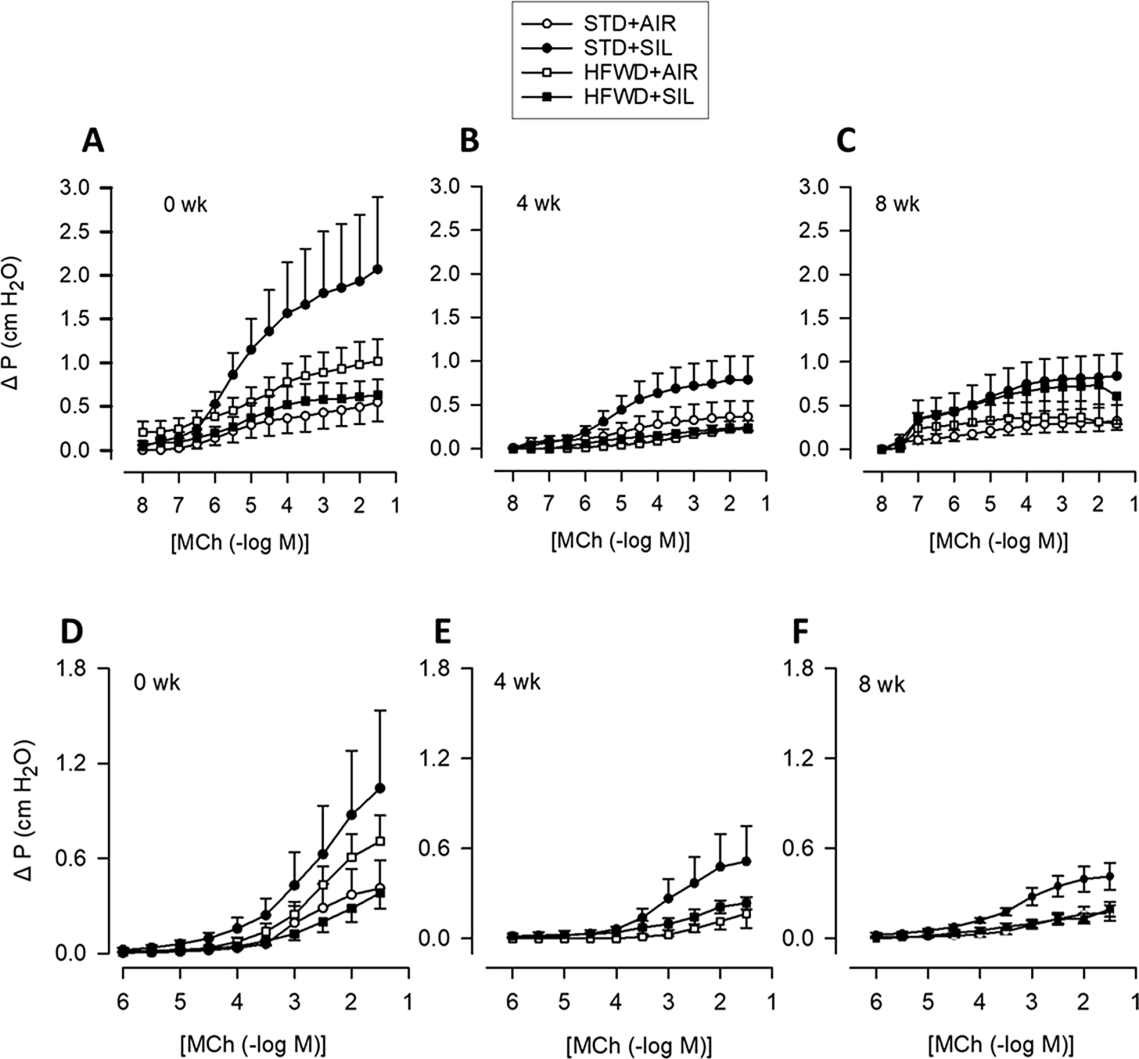
Effect of silica-inhalation, HFWD-consumption, and combined exposure on airway reactivity to applied MCh in the isolated perfused trachea preparation. Concentration–response curves for extraluminally applied MCh at **A** 0, **B** 4, **C** 8 weeks and for intraluminally applied MCh at **D** 0, **E** 4, and **F** 8 weeks post-silica exposure. n = 5–6, 4–6, 5–7 at 0, 4, and 8 weeks, respectively

**Fig. 6:**
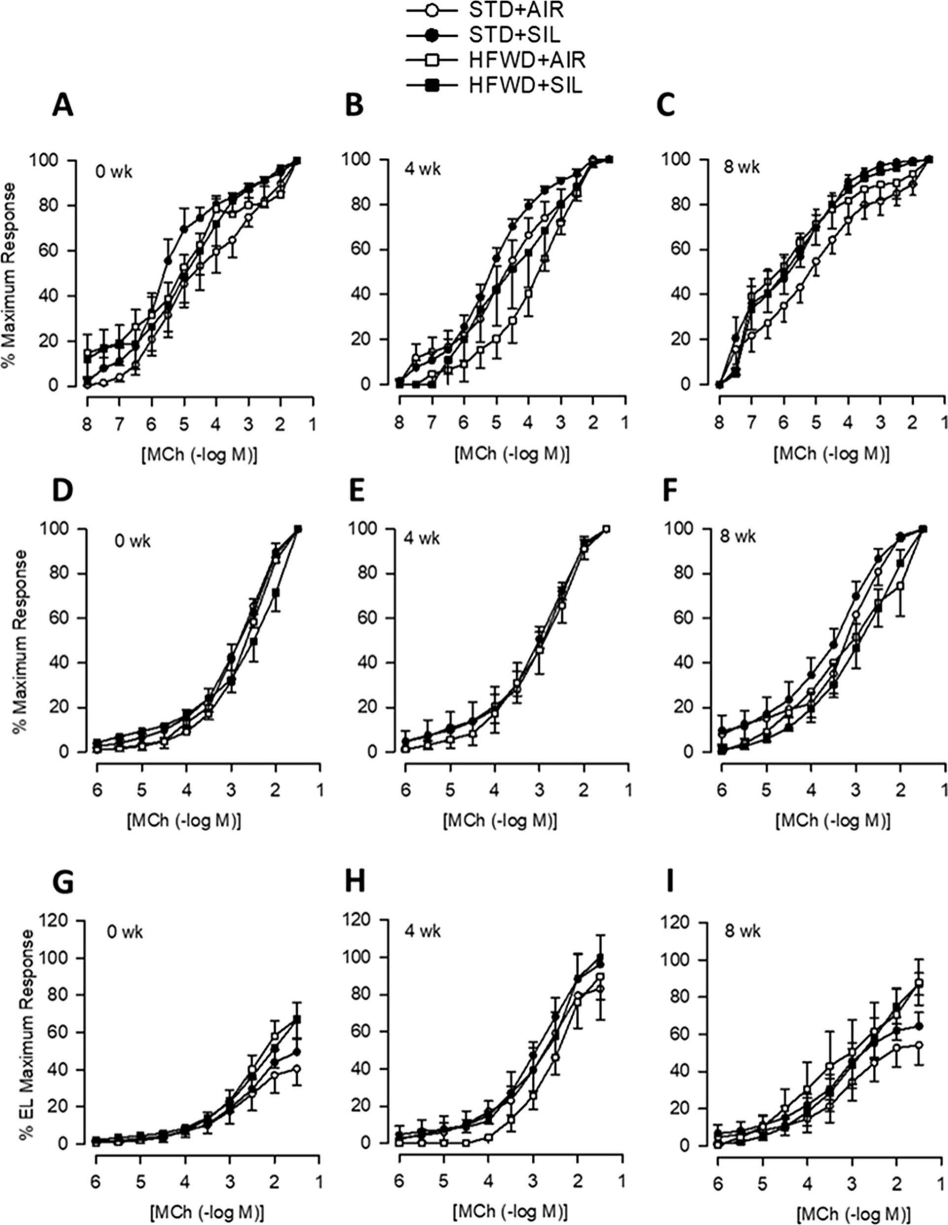
Effect of silica-inhalation, HFWD-consumption, and combined exposure on normalized responses to MCh. Graphs depict percent maximum responses to extraluminally applied MCh at **A** 0, **B** 4, and **C** 8 weeks; intraluminally applied MCh at **D** 0, **E** 4, and **F** 8 weeks; and intraluminal response expressed as a percentage of the extraluminal maximal contractile response to MCh at **G** 0, **H** 4, and **I** 8 weeks post exposure to silica**.** n = 5–6, 4–6, 5–7 at 0, 4, and 8 weeks, respectively

MCh response curves were utilized to determine changes in airway smooth muscle reactivity and epithelial modulation of ASM responses. While we found a trend of increased contractile responses in silica exposure groups, these responses were not statistically significant (Fig. 5). In fact, there were no significant differences in MCh-induced ASM contractile responses (EL), airway epithelium-modulated contractile responses (IL) (Figs. 5, 6), or EC50 values (Table 2) between any of the groups at a given timepoint. Our results suggest that HFWD consumption, silica exposure, and combined exposure (HFWD + SIL), do not significantly alter smooth muscle reactivity or epithelial modulation of the ASM.

**Table 2:** EC50 values from isolated, perfused trachea experiments

Exposure	EC50 [− log (M)]		
	EL	IL	
0 week	Mean ± S.E.M	Mean ±S.E.M	n
STD + AIR	5.98 ± 0.18	2.80 ± 0.17	5
STD + SIL	5.50 ± 0.18	2.63 ± 0.25	6
HFWD + AIR	5.69 ± 0.54	2.81 ± 0.15	6
HFWD + SIL	5.21 ± 0.39	2.37 ± 0.25	5
4 weeks			
STD + AIR	4.98 ± 0.33	2.92 ± 0.18	5
STD + SIL	5.38 ± 0.15	2.88 ± 0.11	6
HFWD + AIR	5.01 ± 0.46	2.35 ± 0.15	4
HFWD + SIL	5.37 ± 1.08	2.64 ± 0.19	4
8 weeks			
STD + AIR	5.50 ± 0.18	3.03 ± 0.18	7
STD + SIL	5.44 ± 0.09	3.20 ± 0.07	5
HFWD + AIR	6.06 ± 0.20	3.76 ± 0.62	5
HFWD + SIL	5.93 ± 0.21	2.97 ± 0.38	6

EC50 values for extraluminal (EL) and intraluminal (IL) concentration–response curves obtained at 0, 4 and 8 weeks post-exposure to silica and or HFWD and air (control). There were no changes in either EL or IL EC50 values in response to silica, HFWD or combined exposure. n values are given for each group

## Discussion

We tested the hypothesis HFWD consumption alters silica’s effect on epithelial ion transport and ASM reactivity, based on our previous findings that HFWD-consumption altered other silica-induced effects in this same animal model [13, 14]. The F344 rat model of silicosis was chosen for use in this study, instead of other animal models, because it is a well-characterized model of silicosis [18, 19]. This model mimics the accelerated type of human silicosis, in which, workers exposed to a large amount of silica over a short period of time develop progressive pulmonary inflammation and fibrosis [18] that continues even after silica exposure has ended [19]. In addition, F344 rats fed HFWD develop metabolic dysfunction similar to diet-induced metabolic disease in humans [14], therefore, this was a good animal model for use in our combined diet and silica exposure studies.

Our results reveal that HFWD-consumption potentiates silica-induced Na^+^ transport at 0 and 4 weeks; these changes were transient and resolved at 8 weeks. In epithelial cells, the Na^+^,K^+^,-ATPase maintains the electrical gradient and movement of Na^+^ ions across the epithelium, which also maintains an ASL depth and composition required for mucociliary clearance of the airways [37]. Insulin’s effects on ENaC regulation in the lung [38, 39] and renal epithelium [40] have been described, and hyperinsulinemia is associated with reduction of Na^+^/K^+^-ATPase activity in rodents and humans [41]. We did not find any change in airway epithelial Na^+^ transport in the HFWD + air group, or changes in Na^+^/K^+^-ATPase, despite elevated insulin levels observed in HFWD + air-exposed animals at 0 and 4 weeks [14], thus indicating the effect observed in the combined HFWD + SIL group was synergistic in nature.

Changes in epithelial Na^+^ transport can impair mucociliary clearance in the airways due to resulting changes ASL depth and cilia beat efficiency, and mucin viscosity and hydration [37]. Studies by Antonini et al. [42, 43] found that rats pre-exposed to silica had enhanced clearance of *L. monocytogenes* from the lung, however, these results were attributed to a silica-induced elevation in immune response including an increased levels of reactive oxygen species [42], increased number of immune cells and activation of alveolar macrophages [43]. Surprisingly, results from those studies are direct opposition of our understanding that silicosis is a well-established risk factor for tuberculosis infection [27].

We propose that the HFWD-induced hyperinsulemia and systemic inflammation [14], were the first-insult that increased epithelial susceptibility to silica-induced damage to airways. Yu et al. [24] demonstrated that silica exposure induces airway epithelial cell injury including the loss of cilia, cilia structure, and mucus hypersecretion which is similar to the atypical cilia [44] and impaired mucociliary clearance mechanisms of silicotic patients [45]. It is plausible that silica-induced ROS reacts with other membrane proteins such as ENaC (the amiloride sensitive Na^+^ channel) or other Na + ion exchangers located on the apical epithelial surface, and in combination with HFWD-induced hyperinsulemia, alter normal ion transport and cellular function. This would also explain the transient nature of our observation; silica particles would be largely cleared from the conducting airways by the 8 weeks post-exposure endpoint, and we found Na^+^ transport returned to normal levels through either recovery or compensatory mechanisms.

It is worthy of mention that Russ et al. [46] found inhalation of fracking sand dust (FSD) for 4 days altered Na^+^ transport in rat airway epithelium and attenuated amiloride’s inhibition of Na^+^ transport, whereas we found that inhalation of silica increased Na^+^ transport. These differences may be attributed to differences in particle composition and size, exposure duration, and cumulative silica exposure. FSD particles consist of a mixture of quartz and other elements with particle size range from 50 µm to 100 nm, while Min-U-Sil 5^®^ particles consist of ≥ 99.5% SiO_2_ and ≥ 5 µm [47]. Nonetheless, it is interesting that both FSD and crystalline silica affected sodium transport.

We found no effect of silica exposure, HFWD-consumption, or the combined exposure, on airway smooth muscle reactivity. Airway hyperreactivity is not greatly associated with silica exposure, however, one study found that a single dose of intranasally-instilled silica (50 mg/0.1 ml/rat) induced airway hyperreactivity to carbachol and 5-HT in tracheal strips 8 weeks post-exposure [48]. We attribute the discrepancy between that study and our own to differences in study design including different rat strains and mode of silica exposure. There is greater evidence associating airway hyperresponsiveness with obesity in humans but with mixed results [49–53]. Orfanos et al. [54] found that human airway smooth muscle (HASM) cells obtained from obese patients exhibited hyperresponsiveness, increased agonist-induced contractility, myosin light chain phosphorylation, and calcium mobilization, compared to HASM cells from non-obese patients. Hyperinsulinemia triggered signaling pathways of cholinergic neurons in the brain stem that can increase airway reactivity in obese mice [33], and it also inhibits neuronal M2 muscarinic receptors, resulting in increased ACh release from airway parasympathetic nerves [32]. While we did not examine effects of HFWD and silica upon neuronal innervation of the airways in our study, the evidence that hyperinsulemia can induce ASM hyperresponsiveness suggests the need for further investigation.

## Limitations and future directions

Limitations of this study include that blood insulin levels were not measured in cohorts of animals used in this specific study. Previously, we found that HFWD-consumption altered serum insulin levels, and our results were similar to findings of other studies where F344 rats were fed a high-fat high-sugar diet [55, 56]. Our interpretation of HFWD-induced hyperinsulemia effects on airway epithelial ion transport rely on the hyperinsulemia data collected from that previous study, in which animals were obtained from the same vendor and were treated identically. A second limitation to this study is that there was no histological investigation conducted on the airway epithelium or airways, although our previous study did use histopathology to identify changes in the lung [13]. Histology may have provided insight into possible morphological changes in connection with our observed changes in ion transport. In addition, investigation of mucociliary clearance of inhaled silica particles in our HFWD + SIL animal model would be tremendously insightful regarding relative risks for infection in those silica-exposed workers with pre-existing MetDys.

Finally, investigation of the potential for HFWD-consumption to alter silica-induced ventilatory pulmonary function responses is needed. Hyperinsulinemia is shown to increase airway hyperreactivity via CNS initiated stimulation of M3 receptors and acetylcholine release in the lung in obese mice [33] and through inhibition of the M2 muscarinic receptor and negative feedback loop that limits acetylcholine release at efferent nerve endings [32, 51]. While we did not detect airway smooth muscle hyperreactivity at the timepoints examined, we previously reported that serum insulin levels increased at 4 weeks in HFWD + air- and HFWD + silica-exposed animals [14], in agreement with other studies reporting hyperinsulinemia in rats fed a high-fat high-sugar diet [52–54]. Investigation of HFWD + silica exposure on neural innervation of the airways and overall pulmonary function is necessary to determine if neural innervation pathways are altered.

## Conclusions

In summary, we found that HFWD-consumption alters silica-induced metabolic responses in airway epithelial Na^+^ ion transport. These changes have the capacity to impair mucociliary clearance mechanisms and suggest a potential increased risk for silica-exposed workers with pre-existing MetDys. In conclusion, these previously unknown interactions between diet and occupational silica exposure are significant and warrant further investigation.

## Data Availability

The original data are available at https://www.cdc.gov/niosh/data/datasets/RD-1068-2023-0/.
